# Exposure to Sublethal Doses of Fipronil and Thiacloprid Highly Increases Mortality of Honeybees Previously Infected by *Nosema ceranae*

**DOI:** 10.1371/journal.pone.0021550

**Published:** 2011-06-28

**Authors:** Cyril Vidau, Marie Diogon, Julie Aufauvre, Régis Fontbonne, Bernard Viguès, Jean-Luc Brunet, Catherine Texier, David G. Biron, Nicolas Blot, Hicham El Alaoui, Luc P. Belzunces, Frédéric Delbac

**Affiliations:** 1 Clermont Université, Université Blaise Pascal, Laboratoire Microorganismes: Génome et Environnement, BP 10448, Clermont-Ferrand, France; 2 CNRS, UMR 6023, LMGE, Aubière, France; 3 INRA, UMR 406 Abeilles & Environnement, Laboratoire de Toxicologie Environnementale, Site Agroparc, Avignon, France; Tulane University School of Public Health and Tropical Medicine, United States of America

## Abstract

**Background:**

The honeybee, *Apis mellifera*, is undergoing a worldwide decline whose origin is still in debate. Studies performed for twenty years suggest that this decline may involve both infectious diseases and exposure to pesticides. Joint action of pathogens and chemicals are known to threaten several organisms but the combined effects of these stressors were poorly investigated in honeybees. Our study was designed to explore the effect of *Nosema ceranae* infection on honeybee sensitivity to sublethal doses of the insecticides fipronil and thiacloprid.

**Methodology/Finding:**

Five days after their emergence, honeybees were divided in 6 experimental groups: (i) uninfected controls, (ii) infected with *N. ceranae*, (iii) uninfected and exposed to fipronil, (iv) uninfected and exposed to thiacloprid, (v) infected with *N. ceranae* and exposed 10 days post-infection (p.i.) to fipronil, and (vi) infected with *N. ceranae* and exposed 10 days p.i. to thiacloprid. Honeybee mortality and insecticide consumption were analyzed daily and the intestinal spore content was evaluated 20 days after infection. A significant increase in honeybee mortality was observed when *N. ceranae*-infected honeybees were exposed to sublethal doses of insecticides. Surprisingly, exposures to fipronil and thiacloprid had opposite effects on microsporidian spore production. Analysis of the honeybee detoxification system 10 days p.i. showed that *N. ceranae* infection induced an increase in glutathione-S-transferase activity in midgut and fat body but not in 7-ethoxycoumarin-O-deethylase activity.

**Conclusions/Significance:**

After exposure to sublethal doses of fipronil or thiacloprid a higher mortality was observed in *N. ceranae*-infected honeybees than in uninfected ones. The synergistic effect of *N. ceranae* and insecticide on honeybee mortality, however, did not appear strongly linked to a decrease of the insect detoxification system. These data support the hypothesis that the combination of the increasing prevalence of *N. ceranae* with high pesticide content in beehives may contribute to colony depopulation.

## Introduction

Honeybee pollination contributes to agriculture productivity and biodiversity. One-third of food consumed in the world is linked to the pollination activity of honeybees, representing a global economic worth of 153 billion euros in 2005 [1]. A significant and poorly understood decrease in honeybee populations, however, has been reported worldwide by beekeepers and scientists. Several factors have been proposed to explain the honeybee decline including nutrition, queen quality, intoxication by pesticides and parasitic diseases [2].

The honeybee, *Apis mellifera* (Hymenoptera, Apoidea), may be exposed to a wide range of pesticides when foraging or consuming contaminated food (pollen and honey) stocked into the hive [3]. Two classes of systemic pesticides, neonicotinoids and phenylpyrazoles, are mainly suspected for negative effects on honeybee health. There are intense debates about the use and the eventual restriction of these pesticides. In many studies, the lack of knowledge about their toxicological profile has prevented drawing conclusions about a causal link between exposure to insecticides and the honeybee decline [4]. This is partly due to the fact that the assessment of the risk posed by pesticides is mainly based on the determination of acute toxicity using LD_50_ as the critical toxicological value [5]. This approach is contested because it cannot account for chronic toxicity and sublethal effects that are highly important elements of neonicotinoid and phenylpyrazole toxicity in honeybees [6]–[8]. Indeed, low doses of neonicotinoids and phenylpyrazoles induce a broad range of sublethal effects such as behavioral or physiological alterations in honeybees and other beneficial arthropods [9].

The adverse effects usually induced by pesticides are limited by the action of a large set of metabolic enzymes. Although honeybees have fewer genes involved in detoxification than other insects [10], they are not necessarily more sensitive to pesticides [11]. In honeybees, detoxification processes occur mainly in both midgut and fat body [12], very similar to those of mammals [13]. Induction of microsomal monooxygenases and glutathione-S-transferase is one of the key mechanisms of insect sensitivity to pesticides [13], [14]. The role of detoxification enzymes, however, is not limited to the protection of insect against the deleterious effects of pesticides. These enzymes are also involved in the metabolism of endogenous compounds such as hormones and pheromones [10]. Therefore, changes in the activity of the detoxification system can lead to variations in honeybee sensitivity to pesticides and more generally to alteration of their physiological homeostasis.

Parasites may also impact insect homeostasis to promote their development. This usually induces changes in insect development, behavior, reproduction and parasite tolerance. Physiological changes induced by parasitism can render insects more susceptible to environmental stressors such as pollutants and may cause a reduction of insect fitness [15]. This trend is particularly exploited in the concept of integrated pest management (IPM) where entomopathogenic parasites are used in association with insecticides at low doses [16]–[18]. Honeybees are also victim to such joint effects between parasites and insecticides. Potential interactions between *Nosema* and pesticides have been firstly described by Ladas in 1972 [19]. More recently, Alaux et al. demonstrated that co-exposure to microsporidian parasites and imidacloprid weakens honeybee [20]. This result corroborates the hypothesis of a multi-factorial cause for the massive colony losses observed worldwide.

Two microsporidian species, *Nosema apis* and *Nosema ceranae*, are the agents of two major diseases known as nosemoses A and C, respectively [21]. Both species are obligate intracellular parasites of adult honeybees. *N. ceranae* increases energetic demand in honeybees [20], [22] and decreases hemolymph sugar level [23]. Furthermore, *N. ceranae* infection significantly suppresses the honeybee immune response [20], [24] and increases ethyl -oleate content (a primer pheromone which regulates worker behavioral maturation) [25]. Finally, *N. ceranae*-infected honeybees have shorter life-spans than uninfected honeybees [20], [26].

There are no data related to the effect of *N. ceranae* on the detoxification system of honeybees. Therefore, it is not possible to consider the link between *N. ceranae* infection, detoxification capacity of infected honeybees and their sensitivity to pesticides. However, we hypothesized that detoxification system could be modified by *Nosema* infection given that detoxification mainly occurs in the gut and that this tissue is the site of *N. ceranae* proliferation. In this study, we assessed the impact of *N. ceranae* infection on detoxification activity of honeybees as well as their sensitivity to fipronil (phenylpyrazole) and thiacloprid (neonicotinoid), two pesticides found at high levels in hives [3]. Based on their oral LD_50_ values, fipronil (LD_50_: 4.17 ng/bee) and thiacloprid (LD_50_: 17 µg/bee) are considered highly and slightly toxic, respectively, to honeybees [27]. We demonstrate, however, that a daily exposure 1/100^th^ concentration of the LD_50_ significantly affects the mortality rate of *N. ceranae*-infected honeybees.

## Materials and Methods

### Experimental procedures and artificial rearing

All experiments were performed with a mixture of honeybees from three Buckfast colonies (crossed with the *Apis mellifera mellifera* honeybee). We used 3 colonies to get sufficient emergent bees for all the experiments (∼2000 bees). We confirmed that these colonies were free of *Nosema* by PCR using primers previously described [26]. Two frames of sealed brood were taken in each colony and placed in an incubator in the dark at 35°C with 80% relative humidity. Emerging honeybees were collected, confined to laboratory cages (Pain type) in groups of 50, and maintained in the incubator for five days. During this time, the caged honeybees were fed with candy (Apifonda®) and water *ad libitum* and were supplied with pollen. To mimic the hive environment as much as possible, a little piece of wax and a Beeboost® (Pherotech, Delta, BC, Canada) releasing a queen's mandibular pheromone, were placed in each cage. After five days of feeding, six experimental groups of individuals were created: (i) uninfected controls, (ii) infected with *N. ceranae*, (iii) uninfected and chronically exposed to fipronil, (iv) uninfected and chronically exposed to thiacloprid, (v) infected with *N. ceranae* and chronically exposed 10 days post-infection (p.i.) to fipronil, and (vi) infected with *N. ceranae* and chronically exposed 10 days p.i. to thiacloprid.

Honeybees were first individually infected (see below honeybee infection) and fed during 10 days with 50% (w/v) sugar syrup supplemented with 1% (w/v) proteins (Provita'bee, Biové laboratory) 10 h per day and thereafter (14 h per day) were fed with candy and water *ad libitum*. Each day, feeders were replaced and the daily sucrose consumption was quantified. Ten days after infection, honeybees were then exposed to fipronil or thiacloprid by ingesting insecticide-containing sugar syrup *ad libitum* (see below exposure to insecticides). Honeybees not exposed to insecticides were fed *ad libitum* with 0.1% DMSO-containing sugar syrup. The feeders were replaced and the daily insecticide consumption was quantified. Throughout the experiment, each cage was checked every morning and any dead honeybees removed and counted.

### Honeybee infection

Spores of *N. ceranae* were obtained from honeybees infected experimentally in our laboratory. After sacrifice, the intestinal tract of infected honeybees was dissected and homogenized in PBS using a manual tissue grinder. The suspension was filtered through No. 1 Whatman mesh and the resulting suspension was cleaned by centrifugation and resuspended in PBS. The spore concentration was determined by counting with a hematocytometer chamber. *Nosema* species was confirmed by PCR according the procedure described by Higes et al. [26].

At 5 days post-emergence, caged bees were starved for 3 h, CO_2_ anaesthetized and spread individually in “infection boxes” consisting of 40 ventilated compartments (3.5×4×2 cm). Each compartment was supplied with a tip containing 125,000 spores of *Nosema ceranae* diluted in 3 µL of water. “Infection boxes” were placed in the incubator and 1 h later, bees that have consumed the total spore solution were again encaged (50 bees per cage). Non-infected bees were similarly treated without *N. ceranae* spores in the water.

### Exposure to insecticides

At 10 days p.i., honeybees were exposed *ad libitum* to fipronil or thiacloprid by ingesting insecticide-containing sugar syrup (50% sucrose solution, w/v) supplemented with 1% protein (Provita'bee, Biové laboratory). Stock solutions of fipronil (1 g/L) and thiacloprid (5.1 g/L) were prepared in DMSO and diluted in sugar syrup to obtain a final concentration of 50% sucrose, 1% protein, 0.1% DMSO and 1 µg/L fipronil or 5.1 mg/L thiacloprid. To expose honeybees to sublethal doses of insecticides, the final concentrations were determined so that honeybees absorbed daily an insecticide quantity corresponding to about 1/100^th^ of the LD_50_. The actual insecticide consumption was quantified by measuring the daily amount of insecticide-containing sugar syrup consumed per bee.

### Preparation of microsomal and cytosolic fractions

Enzyme extraction was performed 10 days p.i. for control and *N. ceranae*-infected honeybee groups. Honeybees were CO_2_-anaesthetized and sacrificed by decapitation, the intestinal tract was dissected, and the midgut was separated from the rectum. Ten abdomens devoid of intestinal tract (containing the fat body) and 50 midguts were pooled in ice-cooled tubes and frozen at −80°C until homogenization. They were homogenized twice at 4°C, (TissuLyser™; Qiagen; 5×10 s at 30 MHz) in extraction buffer (1% KCl, 1 mM EDTA, 1 mM PMSF, 0.1 mg/mL soybean inhibitor and 50 mM Tris-HCl pH 7.6). The homogenates were then centrifuged at 12,000× g for 20 min at 4°C and the resulting supernatants centrifuged at 105,000× g for 60 min at 4°C. The final supernatants (containing GST activity) were frozen at −80°C and the microsomal pellets (containing ECOD activity) were resuspended in 20% glycerol (v/v), 1 mM EDTA, 1 mM PMSF and 100 mM sodium phosphate pH 7.4 and frozen at −80°C until analysis. Protein concentration was estimated for each preparation by the technique of Lowry et al. [28] using a standard curve generated from known amounts (5–35 µg) of BSA.

### 7-ethoxycoumarin-O-deethylase (ECOD) assay

The measurement of 7-ethoxycoumarin-*O*-deethylase (ECOD) activity was done as described by de Souza et al. [29]. Briefly, ECOD activity was measured in midgut and abdomen devoid of intestinal tract (*i.e.* the fat body) by adding 1 mg of microsomal proteins to the reaction mixture containing, 0.5 mM NADP, 5 mM G6P, 10 mM MgCl_2_, 1 U G6PD, 200 µM 7-ethoxycoumarin and 100 mM Tris-HCl pH 7.4. After 30 min of incubation, the reaction was stopped with TCA 60% (p/v) then adjusted to pH 9.0 with 1.6 M glycine-NaOH. The medium was centrifuged at 3000× g for 7 min and the production of 7-hydroxycoumarin was quantified by recording the fluorescence (λ_ex_ 380 nm and λ_em_ 455 nm) with a fluorimeter (SFM 25, Kontron instruments) apparatus. The amount of 7-hydroxycoumarin formed by the ECOD activity assay was determined with a standard curve generated from known amounts (0–100 nM) of 7-hydroxycoumarin.

### Glutathione-S-Transferase (GST) assay

Glutathione-S-Transferase (GST) activity was spectrophotometrically assayed by measuring the conjugation of GSH to 1-chloro-2,4-dinitrobenzene using a method adapted from Habig et al. [30]. GST activity was measured in midgut and abdomen by adding enzymatic extract to the reaction mixture containing 1 mM EDTA, 2.5 mM GSH, 1 mM 1-chloro-2,4-dinitrobenzene and 100 mM Na/K-phosphate pH 7.4. GST activity was quantified by recording the appearance of conjugated product at 340 nm during 5 min. GST activity was calculated using Beer Lambert law with ε_340_ = 9,6 mM^−1^·cm^−1^.

### Statistical analysis

Statistica 7.0 (StatSoft inc., Tulsa, USA) was used for the statistical analysis. The significance thresholds were deemed as significant (*p*≤0.05), highly significant (*p*≤0.01), or very highly significant (*p*≤0.001). Mann-Whitney U Test, a non-parametric test, was used to compare the sucrose and insecticide consumptions at 10 days p.i. Detoxification enzyme activities in both midgut and fat body from control and *N. ceranae*-infected groups were compared by using the Mann-Whitney U Test. The effect of *N. ceranae* infection on honeybee sensitivity from exposure to insecticides was analyzed with a survival analysis taking into consideration all groups followed by a Cox-Mantel test (Life Tables) to determine the significant difference between each group. The Wald-Wolfowitz Runs Test (W-W Runs test) was used to compare insecticide uptake in fipronil only, thiacloprid only, *N. ceranae*-fipronil and *N. ceranae*-thiacloprid groups. A Kruskal-Wallis test combined with a multiple comparison of mean ranks for all groups was used to determine the effect of exposure to insecticides on *N. ceranae* spore production in the digestive tract (*i.e.* midgut and rectum).

## Results

### Honeybee infection

**Figure 1:**
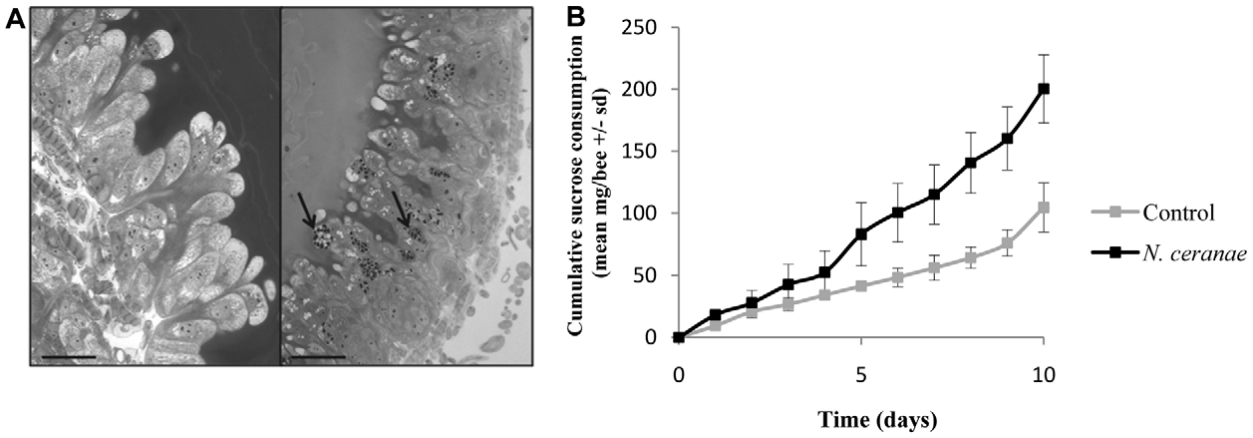
Infection monitoring. (A) Semi-thin sections of midgut epithelium of control (left) and infected (right) honeybees stained with toluidin blue. Arrows indicate *N. ceranae* foci. Bar = 25 µm. (B) Effect of *N. ceranae* infection on sucrose consumption. Data represent the mean of cumulative sucrose consumption (mg/bee +/− standard deviation, sd) from 9 replicates, each containing 50 honeybees.

The success of *N. ceranae* infection was monitored by measuring sucrose consumption of honeybees and counting spores present in their digestive tract 10 days p.i. Light microscopy analysis revealed that numerous *foci* of *N. ceranae* were present at 10 days p.i. in the epithelium cells of infected-bees (**Fig. 1A**). A mean of 18.4.10^6^±0.4.10^6^ spores per honeybee was measured in infected honeybees whereas no spore was observed in uninfected honeybees. PCR analysis confirmed the infection by *N. ceranae* for the infected honeybee group (data not shown). Energetic stress was the main symptom of *Nosema* infection. Thus, the sucrose consumption was compared between infected and uninfected honeybees. For each group (*i.e.* control and infected by *N. ceranae*), the amount of sucrose consumed daily increased with time but differed between these two groups (*i.e.* treatment) (**Fig. 1B**). Indeed, at 10 days p.i., honeybees infected by *N. ceranae* consumed much more sucrose than uninfected honeybees (M-W U test = 2.0, p = 0.0007).

### Changes in detoxification enzyme activity in both midgut and fat body

**Figure 2:**
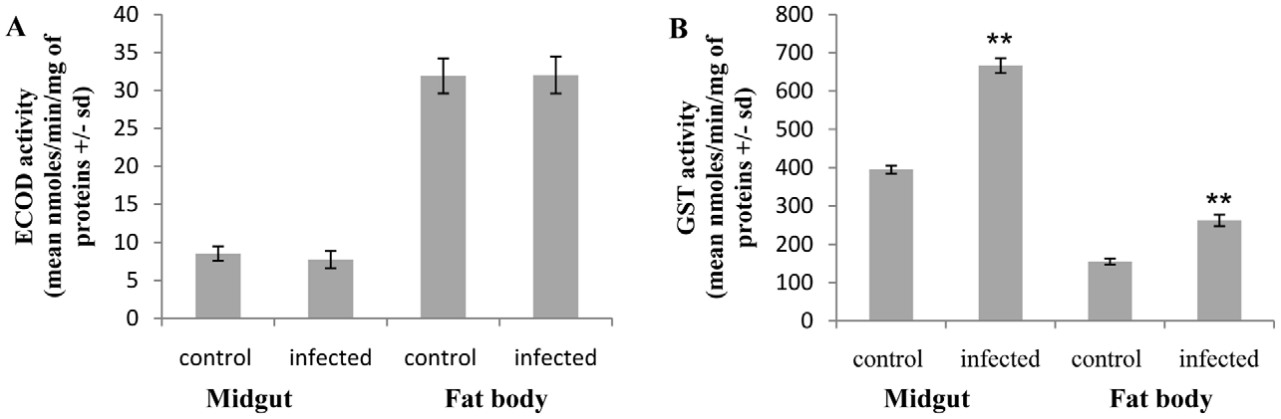
Effect of *N. ceranae* infection on ECOD (A) and GST (B) activities at 10 days p.i. in midgut and fat body of honeybees. Data represent the mean of specific activity (nmoles/min/mg of proteins +/− standard deviation, sd) from 6 replicates (45 honeybees/replicate) of control and infected honeybees. Asterisks indicate the level of significance at p<0.01 (**). ECOD: 7-ethoxycoumarin-O-deethylase; GST: Glutathione-S-Transferase.

ECOD and GST are considered representative of phase I and phase II activities of the detoxification system. The effect of *N. ceranae* on ECOD activity was studied in fat body and mid-gut at 10 days p.i. Infection induced no significant changes in ECOD activity both in midgut (M-W U test = 9.0, p = 0.150) and fat body (M-W U test = 16.0, p = 0.749) (**Fig. 2A**). Conversely, GST activity was highly significantly increased in midgut (M-W U test = 0, p = 0.003 948) and fat body (M-W U test = 0.0, p = 0.003 948) of infected honeybees (**Fig. 2B**). GST activity measured in midgut and fat body of infected honeybees was increased 1.6-fold and 1.7-fold, respectively, compared to the GST activity measured in uninfected honeybees.

### Sublethal doses of fipronil and thiacloprid increases mortality of *Nosema ceranae*-infected honeybees

**Figure 3:**
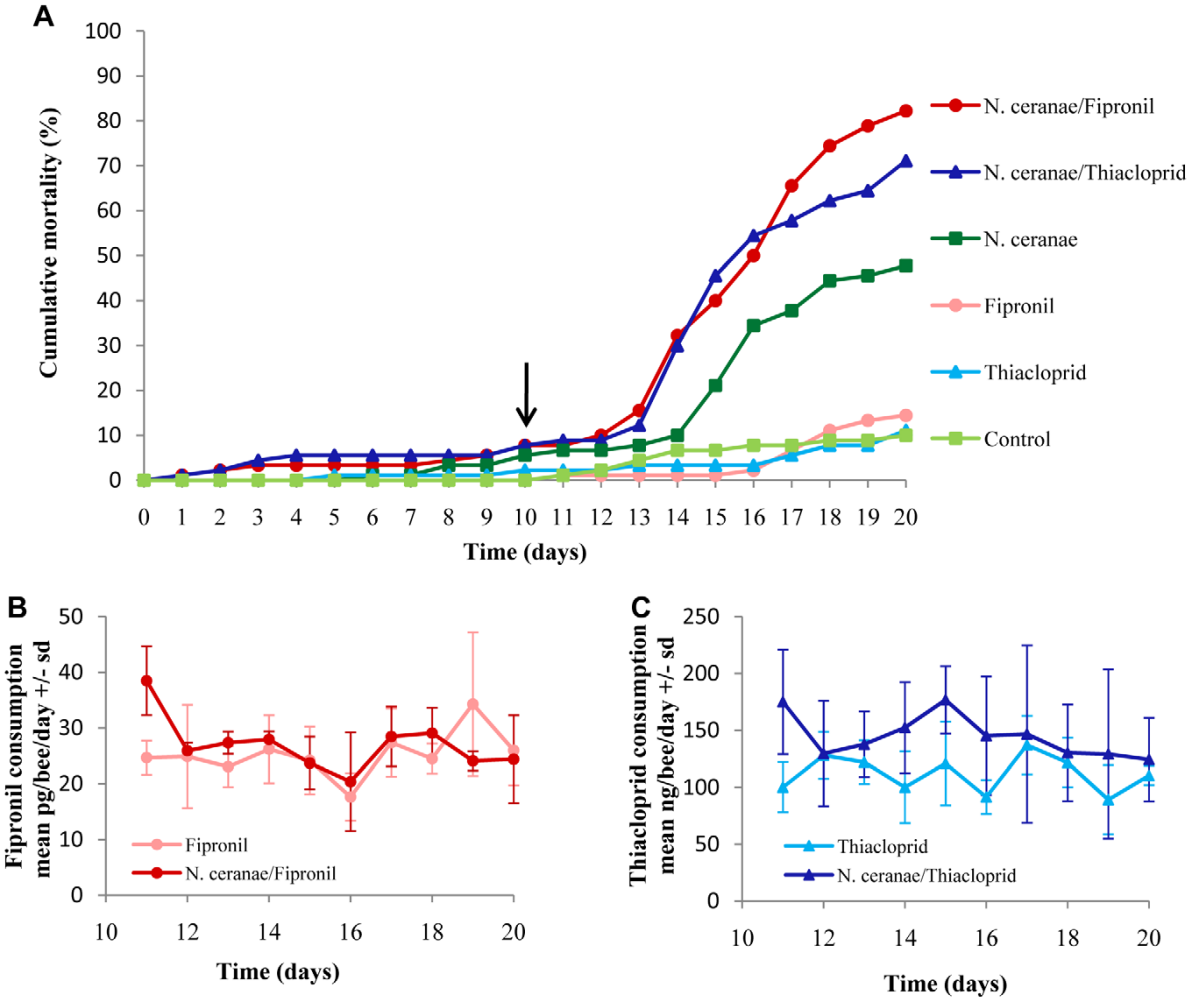
Effect of *N. ceranae* infection on honeybee sensitivity to insecticides. (A) Percentage of honeybee mortality of (i) uninfected control (light green square), (ii) *N. ceranae*-infected (dark green square), (iii) uninfected and chronically exposed to fipronil (light red circle), (iv) uninfected and chronically exposed to thiacloprid (light blue triangle), (v) *N. ceranae*-infected then chronically exposed to fipronil (dark red circle) and (vi) *N. ceranae*-infected then chronically exposed to thiacloprid (dark blue triangle). The arrow indicates the time of exposure to insecticides (10 days p.i.). Data represent the percentage of cumulative mortality calculated from 3 cages, each containing 50 honeybees. The means of fipronil (B) and thiacloprid (C) consumptions (pg or ng/bee/day +/− standard deviation, sd) were daily-monitored until 20 days p.i. for both infected (dark red or blue) and uninfected (light red or blue) honeybees.

The effect of exposure to insecticides on honeybee mortality was assessed in both uninfected and *N. ceranae*-infected honeybees. Survival analysis indicated that *N. ceranae* infection induced a very highly significant increase in honeybee mortality compared to the uninfected control group of honeybees (Cox-Mantel test, U = 31,78, p<10^−5^) (**Fig. 3A**). Exposures to fipronil (Cox-Mantel test, U = 1.87, p = 0.517 68) and thiacloprid (Cox-Mantel test, U = 0.96, p = 0.733 95) had no effect on the mortality of uninfected honeybees compared to uninfected and untreated control group over the duration of our experiments (**Fig. 3A**). Interestingly, honeybee exposure to fipronil (Cox-Mantel test U = 38,41, p<10^−5^) and thiacloprid (Cox-Mantel test U = 28,26, p = p<10^−5^) influenced very highly significantly the amplitude and the time course of *N. ceranae*-induced honeybee mortality (**Fig. 3A**). Indeed, honeybees infected with *N. ceranae* and then exposed to insecticides died earlier than bees only infected. In addition, at the end of the experiment (20 days p.i.), while mortality of only infected honeybees reached a maximum of 47%, the mortalities observed in infected honeybees exposed to fipronil and thiacloprid were not significantly different from each other (Cox-Mantel test U = −10,4864, p = 0.132 65) and reached a maximum of 82 and 71%, respectively (**Fig. 3A**). Comparison of insecticide uptake in fipronil, thiacloprid, *N. ceranae*-fipronil and *N. ceranae*-thiacloprid groups revealed that total consumption of each insecticide at 20 days p.i. was not different in non-infected and *N. ceranae*-infected honeybees for both fipronil (W-W Runs test = 0.46, p = 0,648 08) and thiacloprid (W-W Runs test = 1.37, p = 0,170 90). Also, the daily insecticide uptake was not different in non-infected and *N. ceranae*-infected honeybees for both fipronil (**Fig. 3B**) and thiacloprid (**Fig. 3C**). After exposure to insecticides, uninfected honeybees did not display any signs of intoxication. By contrast, at this level of exposure, insecticides triggered aggressiveness and tremors in infected honeybees during the first days of exposure, and later exhibited ataxia.

### Effect of insecticide exposure on *N. ceranae* spore production

**Figure 4:**
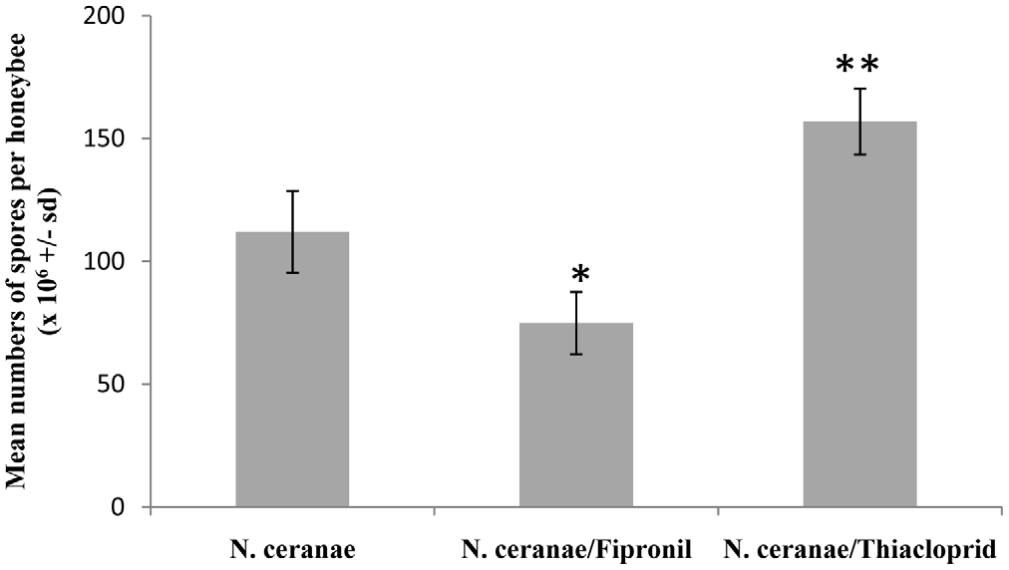
Effect of exposure to insecticides on *N. ceranae* spore production. The spore production in the digestive tract (midgut and rectum) was assessed at 20 days p.i. Data represent the mean numbers of spores/honeybee (×10^6^ +/− standard deviation, sd) from 15 honeybees, 20 days p.i. Asterisks indicate the level of significance at p<0.05 (*) and p<0.01 (**).

Spore production was monitored at 20 days p.i. in all groups (**Fig. 4**). No *N. ceranae* spore was observed in control uninfected bees and insecticide-exposed uninfected honeybees (data not shown). In *N. ceranae*-infected honeybees, a mean of 112.1×10^6^ (±16.7) spores/bee was counted. Interestingly, statistical analysis (Kruskal-Wallis test combined to a multiple comparison of mean ranks for all groups) revealed that exposure to fipronil reduced significantly (p = 0.0117 63) the spore production in infected bees to 74.8×10^6^ (±12.0) whereas exposure to thiacloprid enhanced highly significantly (p = 0.0035, 99) the spore production up to 156.9×10^6^ (±13.3) spores/bee.

## Discussion

In this study, we showed that sublethal doses of a neonicotinoid (thiacloprid) and of a phenylpyrazole (fipronil) highly increased mortality of honeybees previously infected by the microsporidian parasite *N. ceranae*. Although the exact mechanism involved in this synergistic effect remains unclear, our data suggest that the sensitization process is not strongly linked to a decrease of detoxification capacity in infected bees or necessarily by an enhancement of *N. ceranae* proliferation after exposure to insecticides.

During our experiments, no mortality was observed at 10 days p.i. in infected honeybees. Numerous foci were visible in their epithelial cells and a mean of 18.4.10^6^ (±0.4.10^6^) spores/honeybee was measured in their digestive tract. Our results contrast with previous data by Higes et al. [26] who described 100% of honeybee mortality at 8 days p.i. with *N. ceranae*, but are comparable to mortality rate and spore production observed by Paxton et al. [31]. In addition, the very highly significant enhanced sucrose consumption by infected honeybees is consistent with the energetic stress recently described by Mayack and Naugh [22]. Thus, at 10 days p.i., we can consider that the infected honeybees of our study displayed a level of *N. ceranae* invasion seen in forager honeybees [32].

The first evidence of a synergistic interaction between *Nosema* infection and insecticide exposure in honeybees was described by Alaux et al. [20]. These authors demonstrated that *Nosema* spp treatment combined with exposure to imidacloprid, another neonicotinoid, resulted in a higher mortality of honeybees. Based on these results, we hypothesized that *N. ceranae* infection could alter the functioning of detoxification system. We assessed the ECOD and GST activities to test this hypothesis. In insects, ECOD and GST activities have often served as convenient measures of overall phases I and II metabolizing enzyme activities. In addition, levels of ECOD and GST activities have been associated with sensitivity to insecticides [33], [34]. Our results showed that ECOD activity remained unchanged at 10 days p.i, in fat body and midgut whereas GST activity increased significantly in both tissues. Therefore, these data indicated that the higher mortality observed after insecticide exposure in *N. ceranae*-infected honeybees was not strongly linked to a decrease in detoxification capacity. However, we cannot exclude that infection by *N. ceranae* could modify other enzymes involved in detoxification of these insecticides. Despite this observation, exposure to sublethal doses of fipronil and thiacloprid increased the mortality rate in *N. ceranae*-infected honeybees. Indeed, in our experimental procedure, at 10 day p.i. honeybees were chronically exposed to very low doses of insecticides during 10 days. Fipronil consumption was not different in infected and uninfected honeybees. The mean of daily fipronil consumption corresponded to an exposure equivalent to the LD_50_/158 (25.3±4.8 pg/bee) for uninfected honeybees and LD_50_/148 (26.9±0.8 pg/bee) for infected honeybees. A comparable exposure level has been previously considered as sublethal by Aliouane et al. [35]. In the same way, honeybees exposed to thiacloprid consumed a similar daily quantity of insecticides of LD_50_/151 (112.1±4.4 ng/bee) and LD_50_/112 (152.8±8.7 ng/bee) for uninfected and infected honeybees, respectively. As suspected, these levels of fipronil and thiacloprid exposure had no effect on the mortality of uninfected honeybees and on their behavior. Surprisingly, the same levels of exposure caused symptoms of poisoning in infected honeybees and influenced the mortality rate.

Because this metabolic hypothesis failed to explain the sensitization process observed with mortality data, we assessed the effect of exposure to insecticides on spore production. Our results indicated that exposure to fipronil and thiacloprid had antagonist effects on spore production. Indeed, in comparison to infected honeybees not exposed to insecticides, the spore production decreased by about 33% during exposure to fipronil whereas the spore production increased by 40% with thiacloprid exposure. These results then, do not explain the mortality increase observed in the presence of insecticides. First, exposures to fipronil and thiacloprid induced an increase in mortality among infected honeybees but had opposite effects on spore production. Second, in the case of thiacloprid, the spore overproduction did not seem sufficient to explain the enhancement of honeybees' mortality.

The interactive effect seen between *N. ceranae* and insecticides on honeybee mortality was consistent with the observations in honeybees infected with *Nosema* sp and exposed to imidacloprid [20]. While the synergistic effect observed by Alaux et al. [20] seemed to be linked to an increased consumption of imidacloprid by infected honeybees, the synergistic effect observed in our study, however, was not due to increased food intake following infection. These new data on the synergistic action of *Nosema* and insecticides highlight that such interactions are not restricted to neonicotinoids (imidacloprid, thiacloprid) but extend to other classes of insecticides including phenylpyrazoles.

A further generalization of this phenomenon in honeybees exposed to other insecticides would not be surprising. Several classes of chemical insecticides have already shown potency to interact synergistically with entomopathogenic fungi in other insect species. These kinds of combination are commonly used in integrated pest management because they counteract resistance to insecticides of many insects [36] and allow reducing insecticide doses spread in the environment [16], [37], [38]. For instance, organophosphorus compounds (oxydemeton methyl) and pyrethrinoïd (permethrin) insecticides used in combination with *Beauvaria bassiana*, induced a higher impact on *Spilarctia obliqua* [39] and *Anopheles gambiae* [36] survival, respectively, than the use of these control agents alone. In general, the synergistic effect of these combinations appears at insecticide doses considered sublethal to the target insect [17], [40]. As is our study, the major pattern observed with these combinations included an increase in insecticide toxicity (decrease in LD50 or LC50) and a decreased time to onset of insect mortality. This suggests that susceptibility of insects to pesticides is a more complex phenomenon than previously thought. The influence of parasitism in the ecosystem must be considered in toxicological studies. As shown in our study, the use of the LD_50_ as an indicator of systemic insecticide toxicity leads to an underestimation of the deleterious effects induced in infected honeybees. Indeed, we demonstrated that sublethal doses of insecticides highly impacted *Nosema*-infected honeybee mortality. This precaution is important since *N. ceranae* spreads rapidly and can affect more than 80% of honeybee colonies [41].

Numerous examples of interactions between chemicals and pathogens that affect the insect lifespan have been described [15], [17], [39], [42]–[44]. Unfortunately, physiological mechanisms involved in these interactions remain poorly understood and may even appear to be contradictory. One of the current hypotheses explaining the synergistic effect of such combinations suggests that pathogen metabolites may interfere with the detoxification process [36], [45]. Reallocation of insecticide-detoxifying enzymes to counteract parasitic infections possibly reduces the quantity of enzymes available to target insecticides resulting in changes of insecticide toxicokinetics. Thus, it is possible that the synergistic effect results in an effective increase in sensitivity to insecticides in the presence of a proliferating parasite infection. Ironically, the few published data about the effect of parasitism on metabolizing enzymes of insects showed that a large set of parasitic infections could activate several proteins implicated in insect detoxification (*e.g.* CYP's, GST, esterases) [45]–[50]. Consistently, we showed in our study, that 10 days after infection by *N. ceranae*, the GST activity was enhanced in midgut and fat body of honeybees, in agreement with the increase of the antioxidant activity recently described in *Nosema*-infected queens [51]. This result contrasts with the enhancement of infected-honeybees susceptibility to insecticides, suggesting that GST would not be involved in detoxification process of both fipronil and thiacloprid. However, the production of microsomal monooxygenases is an inducible process [52] and it remains possible that induction of detoxification genes in response to exposure to insecticides was prevented by *Nosema* infection. Thus, uninfected honeybees would be able to respond to insecticides by enhancing detoxification process whereas infected honeybees may not. This could explain the symptoms of intoxication observed in infected honeybees.

Curiously, in most studies reporting synergistic effect of fungus/insecticide combination, the impact of exposure to insecticides on parasite virulence was not investigated [36], [39], [40], [43]. In the rare studies addressing this aspect, the parasite virulence was not enhanced by the insecticides. Instead, despite the synergistic effect on insect mortality, it appears that exposure to insecticides tends to decrease germination or proliferation of the fungus [44]. Indeed, insecticides have potential to affect the various developmental stages of entomopathogenic fungi [18], [53], [54] to further justify why studies of compatibility between parasites and insecticides are important for developing IPM applications. In our study, fipronil and thiacloprid have antagonist effect on *N. ceranae* proliferation whereby fipronil decreases slightly spore production in honeybees. This effect can be attributed either to the cytotoxic effect of fipronil on the intestinal epithelium [55], [56] or to its pro-oxidant action [57] that may affect the reproduction cycle of *N. ceranae*, but this assertion should be confirmed by other experiments. In contrast, thiacloprid increased spore production in our study. This result was not consistent with the observations done by Alaux et al. [20] who showed that imidacloprid decreases slightly spore production in honeybees. Thus, in our studies, the synergistic effect of *N. ceranae* infection and exposure to insecticide did not appear to be linked to enhancement of *N. ceranae* virulence by insecticides.

To conclude, our study confirms that interactions between *N. ceranae* and insecticides constitute a significant risk for honeybee health. The increasing prevalence of *N. ceranae* in European apiary combined with the constant toxic pressure undergone by honeybees, appears to contribute to the honeybee colony depopulation. A better understanding of physiological effects induced both by low doses of pesticides and *Nosema* infection seems essential to elucidate the synergistic effects observed on honeybee mortality. The discovery of molecular and cellular mechanisms involved in the adverse effects induced by pathogens and pesticides would confirm the influence of these stressors on honeybee health. In addition, these data provide additional information that will allow a better assessment of risk associated with these stressors and highlight the urgent need of veterinary products for treating nosemosis.
